# Actuation of Flexible Membranes via Capillary Force: Single-Active-Surface Experiments

**DOI:** 10.3390/mi9110545

**Published:** 2018-10-25

**Authors:** Christina Barth, Carl Knospe

**Affiliations:** Department of Mechanical and Aerospace Engineering, University of Virginia, Charlottesville, VA 22904, USA; cab5sf@virginia.edu

**Keywords:** electrowetting, actuation, capillary pressure, lab-on-a-chip

## Abstract

Conventional approaches to microscale actuation, such as electrostatic, have difficulty in achieving large motion at moderate voltages. Recently, actuators relying on the active control of capillary pressure have been demonstrated, with the pressure change caused by electrowetting on a pair of opposing surfaces. In this work, experimental results are presented from five prototype devices in which only a single active surface is used. The results demonstrate that pressure changes induced in a liquid bridge in this manner can produce large deflections (15 μm) of a flexible membrane. Voltages employed in the tests were moderate (≤25 V). The influence of several design variables, such as membrane diameter and thickness, on the membrane deflection are examined. Theoretical predictions are also presented and generally follow the experimental values. Potential sources for the discrepancies between theory and experimental results are discussed. While deflections obtained using a single active surface are not as large as those obtained with two active surfaces, single-active-surface configurations offer a simple route to achieving adequate deflections for lab-on-a-chip microsystems.

## 1. Introduction

For the past decade there has been strong and growing interest in the development of portable lab-on-a-chip (LOC) devices containing tens or even hundreds of independently controlled valves [1]. Actuation of these membrane valves is typically achieved pneumatically, requiring an external pump and a large number of pneumatic tube connections. To relieve the undesirable manufacturing, size, weight, and power characteristics of this approach, researchers have turned to alternative actuation technologies that permit on-chip, electronic control of LOC valves. By and large, the alternative technologies have fallen short, either requiring large voltage/power or yielding inadequate valve deflection (<5 μm) [2,3,4,5]. The authors have recently presented results showing that large deflections of a flexible membrane, like that appearing in LOC devices, can be achieved via a new actuation technique based upon actively controlling capillary pressure [6]. In this paper we offer new experimental results demonstrating that a simpler actuator design can also achieve substantial membrane deflections, although not as large as those previously reported (>50 μm) [6]. The membranes tested herein have similar thicknesses and diameters as those reported in the literature. However, significantly larger membrane deflections are achieved at much lower voltages.

**Figure 1:**
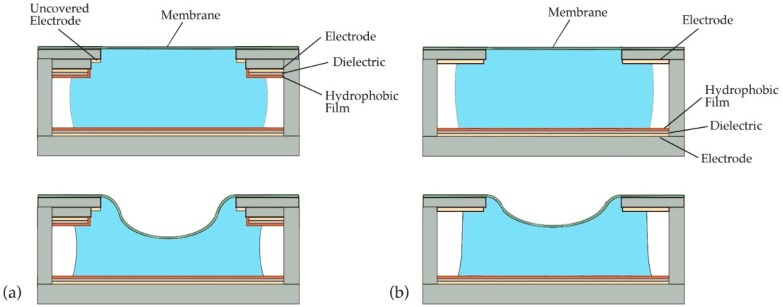
Capillary pressure actuation of a membrane: (**a**) previously investigated dual-active-surface device, before and after application of voltage; (**b**) single-active-surface device examined in this paper, before and after application of voltage. Electrowetting only occurs on the lower surface, not on the upper electrode. This bare electrode can alternatively be placed on the lower surface without changing actuator function.

In both our previous work and that reported on here, membrane actuation is achieved by lowering the capillary pressure of a liquid droplet that is in contact with the membrane. This reduction in pressure is accomplished via electrowetting. In electrowetting, a droplet in contact with a dielectric-covered electrode undergoes a reduction in its contact angle when a voltage is applied between the droplet and the electrode. The change in contact angle alters the curvature of the droplet’s free (i.e., liquid/air) interface and results in a decrease in capillary pressure. In our previous design, electrowetting occurred on two opposing electrodes (see Figure 1a), which we refer to as active surfaces. In this paper, we introduce an alternative membrane actuator containing only a single active surface (see Figure 1b). Such a configuration may offer easier integration into LOC systems since electrical connections only need to be supplied to a single substrate.

## 2. Background

The change in contact angle with applied voltage during electrowetting is governed by the Lippmann-Young equation [7]

(1) $$\cos\left(\theta_{A}\right)=\cos\left(\theta_{A,0}\right)+\frac{\varepsilon_{d}}{2\sigma_{gl}t_{d}}V_{d}^{2}$$

where *ε_d_* is the permittivity of the dielectric film, *t_d_* is the thickness of the dielectric film, $\theta_{A}$ is the contact angle when a voltage $V_{d}$ is applied across the dielectric film, $\theta_{A,0}$ is the contact angle without an electric field, and $\sigma_{gl}$ is the surface tension of the gas/liquid interface. As Equation (1) indicates, greater changes in contact angle will occur during electrowetting when the permittivity is large and the film thickness is small.

Beyond a certain voltage $V_{s}$, known as the saturation voltage, the contact angle ceases to decrease during electrowetting [7,8] and Equation (1) is no longer accurate. The minimum contact angle seen, $\theta_{s}$, will be referred to as the saturation angle. The saturation phenomenon is not fully understood at this time and several causes have been proposed [8]. The thin dielectric films used are exposed to very high electric fields during electrowetting and there may be significant charge injection at larger voltages, even those below the saturation voltage [8,9].

In the membrane actuator considered (Figure 1b), the droplet spans between two surfaces, one of which carries the elastic membrane. The contact angles on the two surfaces will, in general, be different and will vary during actuator operation. These contact angles will be denoted $\theta_{A}$ (active) and $\theta_{P}$ (passive). Electrowetting occurs only on the active surface and the contact angle changes with voltage as described above. On the passive surface, the contact angle is determined by surface and liquid properties. Ideally, the angle on this surface, $\theta_{P}$, does not change ($\theta_{P}=\theta_{P,0}$) when electrowetting occurs on the active surface. In practice, $\theta_{P}$ will vary within a range as determined by the passive surface’s contact angle hysteresis.

**Figure 2:**
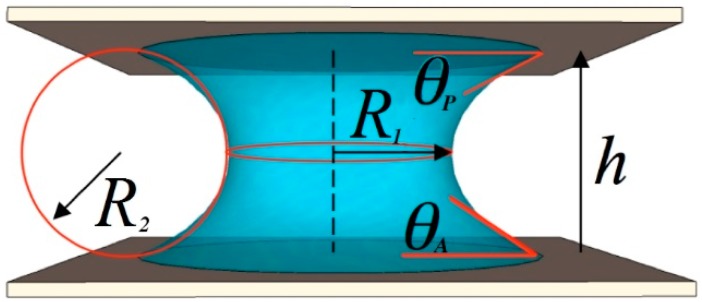
Capillary bridge between surfaces indicating radii of curvature and contact angles.

The liquid bridge that spans between the surfaces will take on an axisymmetric shape that is among the Plateau sequence of shapes [10,11]; this shape is determined by the volume of the bridge, the distance between the surfaces (bridge height, h), and the contact angles [10]. If the radius $R_{1}$ at the bridge’s waist is significantly greater than the bridge height, the profile of the axisymmetric bridge may be accurately approximated by a circular arc [10], whose radius of curvature is denoted $R_{2}$, see Figure 2. This radius of curvature can be expressed in terms of the contact angles [10] as:

(2) $$R_{2}=\frac{-h}{\cos\left(\theta_{A}\right)+\cos\left(\theta_{P}\right)}$$

(A negative radius of curvature corresponds to a bridge profile that is concave.)

The Laplace equation describes the relationship between the bridge’s radii of curvature and the capillary pressure difference ($P_{c}$) across the liquid/air interface:

(3) $$P_{c}=\sigma_{gl}\left(\frac{1}{R_{1}}+\frac{1}{R_{2}}\right)$$

Before voltage is applied, the contact angles of the active and passive surfaces are $\theta_{A,0}$ and $\theta_{P,0}$, respectively. From Equations (1) and (2), the capillary pressure without applied voltage is

(4) $$P_{c,0}=\sigma_{gl}\left(\frac{1}{R_{1}}-\frac{\cos\left(\theta_{A,0}\right)+\cos\left(\theta_{P,0}\right)}{h}\right)$$

After application of voltage to the device and the onset of electrowetting the bridge’s capillary pressure will change to

(5) $$P_{c}=\sigma_{gl}\left(\frac{1}{R_{1}}-\frac{\cos\left(\theta_{A}\right)+\cos\left(\theta_{P}\right)}{h}\right)$$

(Note that the radius of the bridge’s waist, $R_{1}$, remains almost constant during electrowetting as it is much larger than the bridge’s height. For this reason, Equations (4) and (5) use the same value of $R_{1}$) Thus, the change in capillary pressure due to the application of the voltage is given by

(6) $$\Delta P_{c}=\frac{\sigma_{gl}}{h}\left\{\left(\cos\left(\theta_{A,0}\right)-\cos\left(\theta_{A}\right)\right)+\left(\cos\left(\theta_{P,0}\right)-\cos\left(\theta_{P}\right)\right)\right\}$$

When the contact angle on the active surface is lowered via electrowetting, the capillary pressure of the liquid is reduced ($\Delta P_{c}<0$). This change in the liquid’s pressure results in an inward deflection of the flexible membrane due to its contact with the liquid. The reader will note that Equation (6) predicts that larger changes in capillary pressure will occur for a given change in $\theta_{A}$ if the bridge height is made smaller.

The contact angle on the passive surface may change within the range dictated by the surface’s contact angle hysteresis. Due to volume conservation for the bridge, if the passive surface contact line does not advance sufficiently when electrowetting occurs on the active surface, the passive surface contact angle will increase. This result is due to the requirement that the free interface of the liquid bridge must belong to the Plateau sequence of shapes. Therefore, the term $\left(\cos\left(\theta_{P,0}\right)-\cos\left(\theta_{P}\right)\right)$ is typically positive and tends to counteract the decrease in pressure caused by the change in contact angle on the active surface. If there was no contact angle hysteresis on the passive surface, equilibrium of the forces acting upon the passive surface’s contact line would only occur at a single angle, $\theta_{P,0}$, no matter what angle existed on the active surface. In this (ideal) case, the change in capillary pressure would be

(7) $$\Delta P_{c}=\frac{\sigma_{gl}}{h}\left\{\cos\left(\theta_{A,0}\right)-\cos\left(\theta_{A}\right)\right\}$$

and the maximum possible membrane displacement would occur.

For the devices tested in this paper, the layout of components is as shown in Figure 1b. The flexible membrane is attached to the passive surface substrate. This substrate also carries a bare gold electrode that is in contact with the liquid bridge and is needed for completing the electrowetting circuit. The active surface lies opposite the passive surface. When electrowetting occurs, the pressure in the drop is reduced and the membrane is pulled toward the active surface. The reader should note that many configurations of the components are possible. For example, in microfabricated devices, it is desirable to avoid electrodes on both the substrates shown in Figure 1b because of the difficulties associated with electrical interconnects. Since the location of the bare electrode has no impact upon device performance, this can be achieved by having the substrate that contains the active surface also carry the bare electrode. This was not done in this effort since it was simpler to modify an existing fabrication process than to develop one to permit both electrodes on one substrate. Another configuration of the device could have the active surface substrate carry the flexible membrane. The bare electrode could also be on this substrate or on the accompanying passive surface. Such configurations should have essentially the same performance as that seen in this paper.

## 3. Materials and Methods

### 3.1. Fabrication of Electrowetting Surfaces

E-beam evaporation (CHA Industries, Fremont, CA, USA) was used to deposit a 100 nanometer (nm) thick aluminum (Al) film onto borosilicate glass slides (top plate, 120 μm thick) or soda-lime glass slides (bottom plate, 1.59 mm thick). A 30.6 nm aluminum oxide (Al_2_O_3_) film was grown by anodization (18 V) [12]. An ultrathin (50.8 nm) film of the hydrophobic fluoropolymer CYTOP-809 (Bellex International Corporation, Wilmington, DE, USA) was deposited on the oxide film via spin coating and the slides were then baked at 160° for 1 h [13]. To complete the fabrication of the active surface, a silicon oil mixture (Dow Corning OS-10, OS-20, and OS-30, Krayden, Inc., Denver, CO, USA, 1:8:1 by weight), was applied to the CYTOP and then allowed to evaporate from the surface. This treatment was found to reduce contact angle hysteresis and yield more repeatable electrowetting [13,14]. (Contact angle hysteresis was typically 10° after treatment).

### 3.2. Device Components

Proof-of-concept devices were fabricated using facile, low cost techniques that permitted ease in assembly, disassembly, retesting, and visual observation of the liquid bridge during operation. Each device was composed of a passive top plate with a polydimethylsiloxane (PDMS) membrane, a bottom plate with active surface, and a liquid bridge extending between them—see Figure 1b. While dimensions vary among the devices tested, the working components occupy a region of dimensions approximately 2 mm × 2 mm × 0.5 mm. Devices with smaller dimensions should be equally effective according to theory [13] but were not examined in this initial investigation due to the design constraints imposed by simple fabrication procedures and liquid bridge observation.

### 3.3. Fabrication of Membranes

The elastomeric membrane in each device was fabricated using a Sylgard 184 PDMS kit (Dow Corning, Midland, MI, USA). The base polymer and curing agent were mixed 10:1 by weight and allowed to degas in air for 90 min. The PDMS solution was then deposited onto a poly(methyl methacrylate (PMMA) surface and spun for 60 s at 1100–2200 RPM to achieve the desired final thickness, typically 40 or 50 μm. The coated PMMA was then baked in an oven for 1 h at 70 °C. The cured PDMS was then cut into ~5 mm squares. A Dektak Profilometer system (Bruker, Billerica, MA, USA) was used to measure the thickness of PDMS membrane—see Table 1 for measured values. A titanium flake (~100 nm thick) was transferred to the center of each PDMS square to provide a reflective surface for laser interferometric measurement of membrane displacement during testing.

### 3.4. Fabrication of Device Bottom Plate

The bottom plate was a glass slide with an electrowetting surface fabricated as described above (see Fabrication of Electrowetting Surfaces section).

### 3.5. Fabrication of Device Top Plate

The top plate (passive surface) was composed of a borosilicate glass slide with a gold electrode to act as the cathode for electrowetting. Fabrication was achieved using the following procedure. Holes were cut into each slide via VersaLASER VLS3.50, 50 W CO_2_ laser cutter (Universal Laser Systems, Scottsdale, AZ, USA). The hole defines the perimeter of the free-standing elastomeric membrane. After cutting and cleaning, a 10 nm thick layer of Au was deposited via e-beam evaporation, utilizing a 2.5 nm Ti adhesion layer. An ultrathin film of CYTOP was spin coated onto the Au layer, to allow the eased motion of the conducting drop on the passive surface. The CYTOP film was applied with one spin coat application (30 s, 2000 RPM) of either 0.5% wt. or 1% wt. CYTOP and then baked for 1 h at 160 °C. It was found in earlier testing that such ultrathin films of CYTOP by themselves provided essentially no resistance to the conduction of current, undergoing electrical breakdown as soon as any voltage was applied. Thus, the presence of the CYTOP does not affect the prototype’s electrical performance and electrode prepared in this fashion can be considered ‘bare’ in spite of the CYTOP film. The CYTOP layer was then treated with silicon oil as described above. A PDMS piece was then applied to the top of the slide covering the hole with the reflective flake centered within the hole. The PDMS square adhered to the glass with suitable strength and did not require any additional treatment (e.g., oxygen plasma bonding).

Plastic microbeads (Polyscience, Warrington, PA, USA) were deposited from solution onto the lower surface of the top plate to act as spacers and thus establish the distance between the top and bottom plates (i.e., bridge height) in the final assembled device. The beads deposited were (nominally) 100 μm in diameter (±10 μm).

### 3.6. Device Assembly

A “flip chip” assembly process was used to form the test articles. A 0.1M Cs_2_SO_4_ aqueous solution was prepared with deionized (DI) water and 10 μMol/L Fluorescein salt was added (chemicals from Sigma Aldrich, St. Louis, MO, USA). A 1.2–1.5 μL droplet of the solution was deposited within the hole in the top plate (beneath the PDMS membrane) by micropipette (Eppendorf, Hamburg, Germany). Variations in droplet volume will have very little effect on device performance as the capillary pressure is not strongly dependent on $R_{1}$ when $R_{1}\gg h$. The top plate was then placed on the bottom plate with tweezers. The assembled components were held in place by the attraction between a permanent magnet placed below the bottom plate and ferromagnetic foil pieces secured around the PDMS square on the upper surface of the top plate via tape. Due to variations in the size of beads used for spacers (±10 μm) and bow in the glass slides, the bridge height achieved after assembly could not be tightly controlled. Therefore, the height was measured via the side view camera to determine an accurate value for use in theoretical predictions. The side-view camera was also used to detect misalignments between plates. Since the variation in bead diameters was much less than the distance between bead locations, the alignment between the top and bottom plates was very good. When misalignment was detected, the device was reassembled to eliminate it.

### 3.7. Device Testing

A fiber optic extrinsic Fabry-Perot interferometer (Fiber Pro2, Luna Innovations, Roanoke, VA, USA) was employed to measure membrane displacement during testing. The fiber was centered over the reflective flake on the PDMS membrane, brought into range, and the interferometer settings were adjusted to maximize signal quality. The received signal typically temporarily lost quality when displacements were large, and changes were sudden. As a result, spurious spikes sometimes would appear in the displacement data.

The profile of the liquid bridge was imaged via a side view camera (Prosilica GC2450, Allied Vision Technologies, Stadtroda, Germany) paired with a long-distance microscope (K2/SC, Infinity-USA, Centennial, CO, USA) to achieve an effective magnification of ~0.7 μm/px. The inclusion of a fluorescent dye in the liquid allowed imaging of the bridge profile in spite of the close spacing between top and bottom plates of the device. The bridge’s fluorescence was excited using a 405 nm wavelength laser pointer and a bandpass optical filter was placed in front of the aperture of the side view camera to attenuate light at the excitation wavelength.

Only direct current (DC) voltages were used in testing. As discussed below, the anodic oxide dielectric films employed in our devices fail under reversed polarity [12] and preclude alternating current (AC) operation.

## 4. Results

### 4.1. Description of Tests

We highlight the results of five tests here; for further results see [13]. For all the prototypes discussed, bridge heights were set via the microbeads to be close to 100 μm. Membranes were constructed in two sizes, small diameter (~440 μm) and large diameter (~510 μm), and two ranges of thickness, thin (~40 μm) and thick (~50 μm). The relevant dimensions for each prototype device and the testing conditions are described in Table 1. The measured data from these tests as well as calculated values based upon this data are provided in Table 2. The contact angles of the liquid bridge on the passive and active surfaces are given for the time before application of voltage (‘initial’) and for that when the voltage first reaches its peak value (‘final’). Measurements of membrane displacements are also provided for these times. (The values reported are calculated from an average of 10 measurements taken after the transient has settled). The model relating capillary pressure to membrane deflection that was used in this research is contained in the Appendix of our previous work [6]. Theoretical values of membrane displacement were calculated using Equation (6) and are presented in Table 2. Note that these calculations are based upon the measured values of contact angle, not angles derived from the Lippmann-Young equation. Calculated values of the change in capillary pressure between the ‘initial’ state and the ‘final’ state are also displayed in the table. Two values are calculated: (1) a predicted pressure change based on the measured values of contact angle using Equation (6); and (2) a ‘no hysteresis’ case where the pressure change is based on the measured initial and final contact angles on the active surface and the specification that the contact angle on the passive surface does not vary from its initial value (see Equation (7)).

Good agreement between theory and experiment occurred for most tests that were conducted. The expected trends were also apparent in the experimental results. Thin membranes, large diameter membranes, and higher voltages yielded larger membrane deflections. Each test is discussed in detail below.

### 4.2. Staircase Voltage Signal Tests

**Figure 3:**
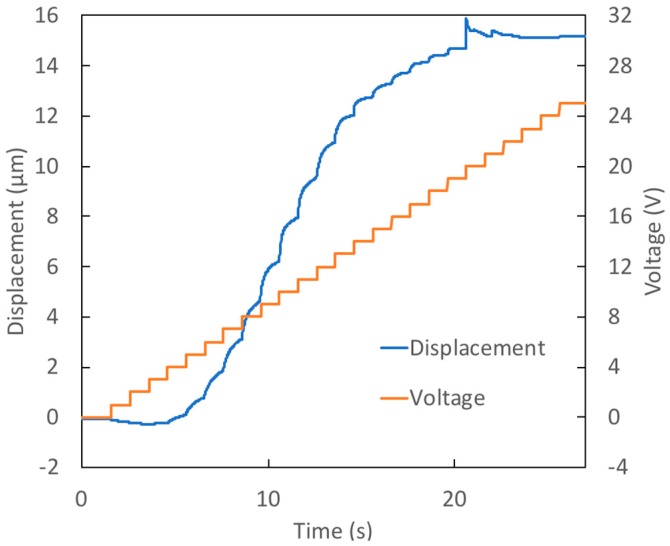
Test 1—Response of prototype with a thin (39 μm), large diameter (510 μm) membrane to staircase voltage signal.

In Test 1, the prototype was a device with a thin, large diameter membrane. In the test, the voltage was increased in 1 V increments each second, stepping from 0 V up to 25 V (a ‘staircase’ signal). Figure 3 shows the measured membrane displacement and voltage during the test. The membrane reaches its maximum displacement, 15.2 μm, when 20 V is applied. For voltage increases beyond this value, the deflection remains essentially constant due to the onset of contact angle saturation. The reader will note that 13 μm (85% of the final deflection) was achieved when only 14 V was applied. The measured deflection is quite close to that predicted from the measured contact angles, exceeding the theoretical prediction by just 10%.

In Test 1, the contact angle on the active surface decreased by 18° between initial and final states. The contact angle on the passive surface, however, increased by 6°. This increase results in less curvature of the interface profile than would occur if the passive surface contact angle remained unchanged. As a result, 40% less change in the capillary pressure occurred in this test than would have taken place in the ‘no hysteresis’ case. This example shows the importance of the passive surface characteristics to achieving good performance.

**Figure 4:**
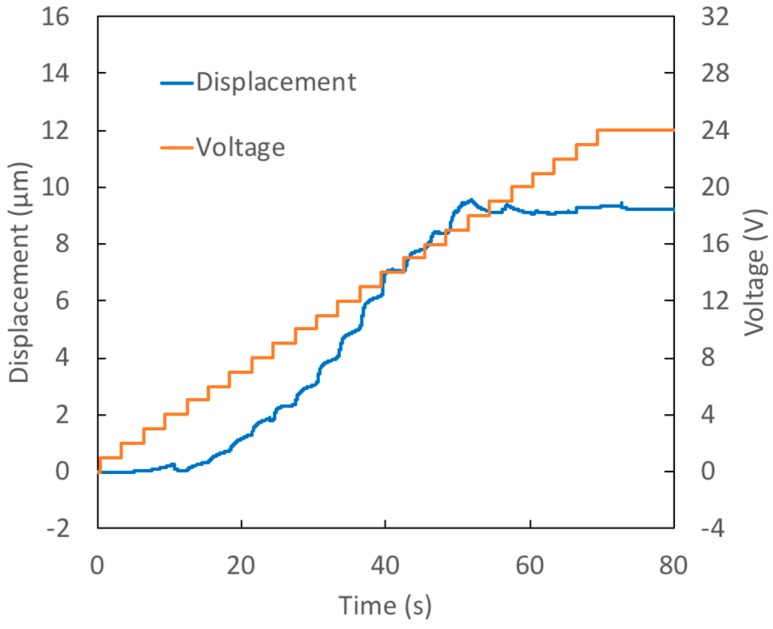
Test 2–Response of prototype with a thin (39 μm), small diameter (439 μm) membrane to staircase voltage signal.

Figure 4 shows the results from Test 2 where a thin membrane with a small diameter was used. The voltage applied was a staircase signal with 1 V steps occurring every 3 s. The maximum voltage applied was 24 V. The changes in the contact angles on the active and passive surfaces are quite similar to those seen in Test 1. As a result, the changes in capillary pressure are also comparable. The reduced deflection seen in Test 2 is therefore primarily due to the smaller diameter membrane employed in this test. Membrane deflection is quite sensitive to this variable: a 14% reduction in membrane diameter results in more than a 50% decrease in displacement.

**Figure 5:**
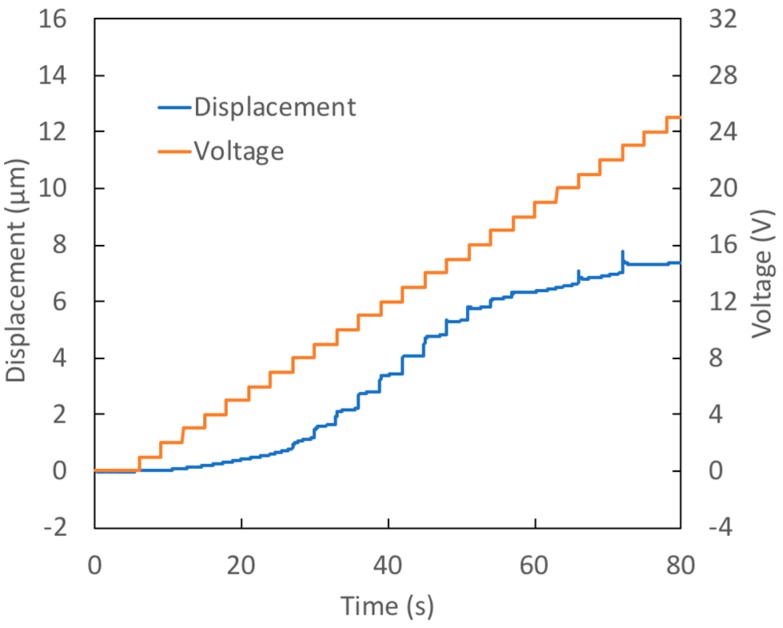
Test 3—Response of prototype with a thick (50 μm), large diameter (510 μm) membrane to staircase voltage signal.

In Test 3, the prototype examined contains a membrane with the same large diameter as used in Test 1, but with 25% greater thickness than that test. The voltage applied was a staircase signal with a maximum voltage of 25 V. The deflection observed in this test is 40% lower (see Figure 5) than that seen in Test 1, primarily due to the thicker membrane used. However, the measured deflection also significantly underperforms the theoretical value. It is likely that this is the result of contact angles that are not as favorable as those that were measured. This circumstance can occur if the bridge is not axisymmetric. The contact angles can vary along the contact line; the angles measured may not be representative of the actual surface curvatures and the capillary pressure that arise from them.

The reader will note that in all three tests the displacement for the most part reaches its maximum by 18 V. This is due to contact angle saturation.

### 4.3. Square Wave Voltage Signal Tests

**Figure 6:**
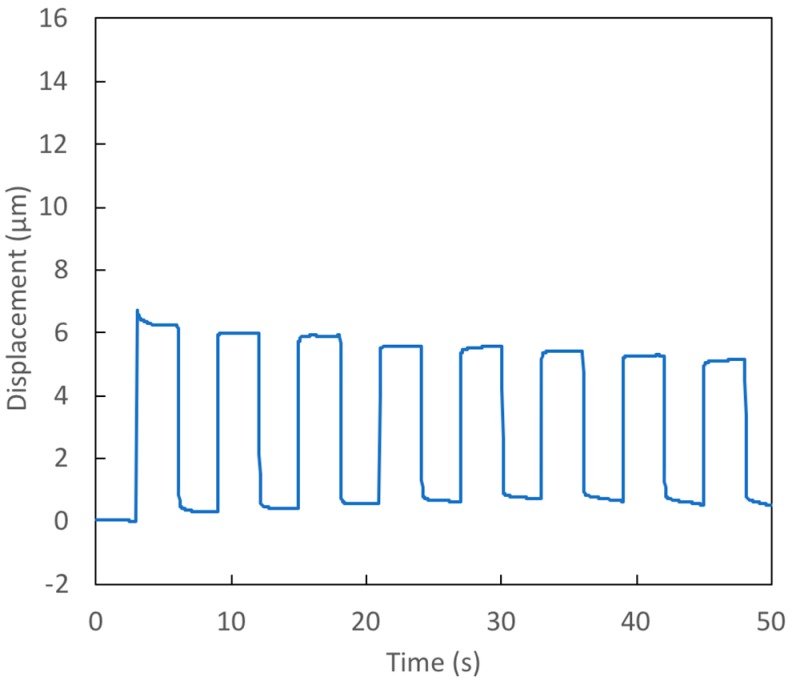
Test 4—Response of prototype with a thin (39 μm), small diameter (438 μm) membrane to 14 V square wave voltage signal.

**Figure 7:**
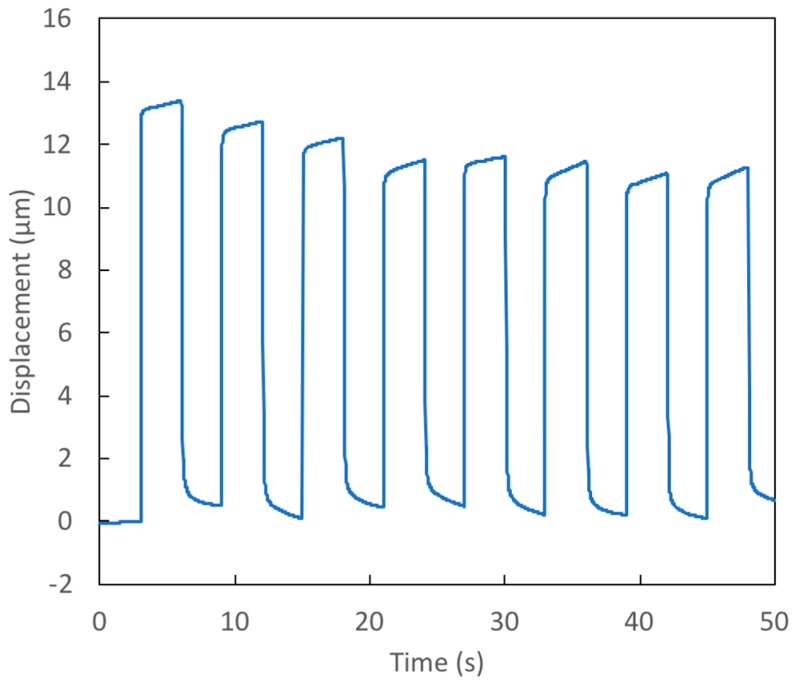
Test 5—Response of prototype with a thin (40 μm), small diameter (441 μm) membrane to 14 V square wave voltage signal.

The prototypes examined in Test 4 and Test 5 are essentially the same as that studied in Test 2; they have a thin, small diameter membrane. The voltage applied in both tests was a square wave with a minimum of 0 V and a maximum of 14 V. The period of the voltage signal is 6 s. Figure 6 and Figure 7 show the measured displacements for Tests 4 and 5, respectively. In spite of the nearly identical dimensions, the deflection seen in Test 4 is significantly less than that in Test 5. The cause of this marked difference can be found in the data in Table 2: the initial contact angle on the active surface was much lower in Test 4 (91°) than in Test 5 (103°). Since the final (electrowetted) contact angle for both tests is essentially the same (~80°), the angle change in Test 4 is much less than in Test 5. Thus, the change in capillary pressure in Test 4 is less than half that seen in Test 5. Since the active surface is coated with CYTOP and treated with silicon oil, we would not expect the sessile angle of the bridge to be as low as 91°. It is likely that there was some contamination in the manufacture of this surface. The result shows that this approach to actuation is quite sensitive to variations in these surfaces. For successful application of this actuator technology, careful attention must be paid to manufacturing processes to ensure quality and consistency. 

Note that in both Test 4 and Test 5, the contact angle on the passive surface changed very little from its initial value during electrowetting. As a result, the capillary pressure change achieved is not far from that which would occur in the ideal ‘no hysteresis’ case. Equation (6) shows that the value of the passive surface’s sessile contact angle has very little effect on actuator performance [10]. The important factor for performance is whether this angle remains unchanged when electrowetting occurs on the active surface, or whether this angle increases.

As seen in Figure 6 and Figure 7, the response time when changing membrane deflection is quite quick, with almost all of the deflection taking place in less than 0.2 s.

## 5. Discussion

As the results demonstrate, capillary pressure changes due to electrowetting are an effective means for the generation of membrane movement in microdevices, even when electrowetting occurs on only one surface and not that containing the membrane. The displacements obtained (5–15 μm), like those found in a previous investigation of dual-active-surface devices [6], were significantly larger than those earlier achieved by electrostatic actuation using much higher voltage and power [4]. This paper’s single-active-surface results opens up a range of possible variations in actuator design. Membranes can be attached to surfaces, either active or passive, in a variety of ways and can adjoin the droplet(s) in many arrangements [15].

The membrane displacements obtained for single-active-surface devices are about half of those obtained by their dual active surface counterparts [6], as predicted by theory [10]. Just as experiments with those actuators demonstrated, significant improvements in the deflection performance of single-active-surface devices can be achieved by decreasing bridge height. With a height of 40 μm, it should be possible to achieve 15 μm of membrane displacement using only 10 V. Thus, the range of motion desirable for LOC applications can be achieved with membranes of typical LOC diameter and thickness.

Experimental results show that contact angle hysteresis on the passive surface is an important factor in affecting actuation performance for single-active-surface devices. If the angle on the passive surface increases significantly as electrowetting occurs, the change in capillary pressure will be substantially reduced. It is therefore desirable to engineer the passive surface to have as little contact angle hysteresis as possible. This would be a fruitful area for further research.

Although a systematic investigation of long-term actuation behavior was not carried out, several comments on this aspect of performance during the square wave tests are warranted. There was some decrease in motion within the first few cycles. If the voltage was less than 14 V, the displacement behavior settled into a steady pattern with an increasing number of cycles and further degradation was not noted. In this case, the magnitude of the displacement motion was between 60% and 80% of the initial displacement, depending upon the test. Charge injection was detected in tests where the voltage exceeded 16 V.

## Figures and Tables

**Table 1:** Dimensions and descriptions for the single-active-surface tests conducted.

Test	Bridge		Membrane		Max Voltage (V)	Description
	Height (μm)	Radius (mm)	Thickness (μm)	Radius (μm)		
1	93	1.36	39	510	25	1 V step staircase
2	94	1.80	39	439	24	1 V step staircase
3	96	1.33	50	510	25	1 V step staircase
4	108	0.97	39	438	14	14 V square wave
5	111	1.23	40	441	14	14 V square wave

**Table 2:** Results from single-active-surface tests—contact angles on active and passive surfaces as measured by side view camera; measured membrane deflection and theoretical value calculated from measured angles; values of change in capillary pressure as determined from measured angles (Equation (6)) and with a no hysteresis model (Equation (7)).

Test	Contact Angles (°)				Maximum Membrane Deflection (μm)			Calculated Capillary Pressure Change, ∆P_c_ (Pa)	
	Initial		Final *						
	$\theta_{A,0}$	$\theta_{P,0}$	$\theta_{A}$	$\theta_{P}$	Measured	Theory	Error (%)	Equation (6)	Equation (7)
1	107	58	89	64	15.2	13.8	10.1	160	227
2	101	89	83	96	7.3	9.0	18.9	138	226
3	104	86	80	94	9.2	12.3	25.2	196	294
4	91	101	81	104	6.2	4.7	31.9	77	110
5	103	94	80	98	13.4	12.2	9.8	202	244

* Final indicates at the time when maximum voltage is first reached.
